# Stimulation TcPO2 Testing Improves Diagnosis of Peripheral Arterial Disease in Patients With Diabetic Foot

**DOI:** 10.3389/fendo.2021.744195

**Published:** 2021-12-10

**Authors:** Vladimíra Fejfarová, Jiří Matuška, Edward Jude, Pavlína Piťhová, Milan Flekač, Karel Roztočil, Veronika Wosková, Michal Dubský, Alexandra Jirkovská, Robert Bém, Jitka Husáková, Věra Lánská

**Affiliations:** ^1^ Diabetes Centre, Institute for Clinical and Experimental Medicine, Prague, Czechia; ^2^ MATMED, Vascular Outpatient Clinic, Hodonín, Czechia; ^3^ Tameside Hospital NHS Foundation Trust, Ashton-under-Lyne, United Kingdom; ^4^ Diabetes Centre, Second Faculty of Medicine, Motol Teaching Hospital, Prague, Czechia; ^5^ First Faculty of Medicine, Charles University, Prague, Czechia; ^6^ Department of Transplant Surgery, Institute for Clinical and Experimental Medicine, Prague, Czechia

**Keywords:** diabetic foot, PAD - peripheral arterial disease, microcirculation, TcPO2 and TcPCO2 measurement, diagnosis

## Abstract

**Background:**

All diagnostic procedures of peripheral arterial disease (PAD) in diabetic foot (DF) are complicated due to diabetes mellitus and its late complications.

The aim of our study is to enhance diagnosis of PAD using a novel transcutaneous oximetry (TcPO2) stimulation test.

**Methods:**

The study comprised patients with mild-to-moderate PAD(WIfI–I 1 or 2) and baseline TcPO2 values of 30-50 mmHg.TcPO2 was measured across 107 different angiosomes. Stimulation examination involved a modification of the Ratschow test. All patients underwent PAD assessment (systolic blood pressures (SBP), toe pressures (TP), the ankle-brachial indexes (ABI) and toe-brachial indexes (TBI), duplex ultrasound of circulation). Angiosomes were divided into two groups based on ultrasound findings: group M(n=60) with monophasic flow; group T(n=47) with triphasic flow. Large vessel parameters and TcPO2 at rest and after exercise (minimal TcPO2, changes in TcPO2 from baseline (Δ,%), TcPO2 recovery time) measured during the stimulation test were compared between study groups.

**Results:**

During the TcPO2 stimulation exercise test, group M exhibited significantly lower minimal TcPO2 (26.2 ± 11.1 vs. 31.4 ± 9.4 mmHg; p<0.01), greater Δ and percentage decreases from resting TcPO2 (p=0.014 and p=0.007, respectively) and longer TcPO2 recovery times (446 ± 134 vs. 370 ± 81ms;p=0.0005) compared to group T. SBPs, TPs and indexes were significantly lower in group M compared to group T. Sensitivity and specificity of TcPO2 stimulation parameters during PAD detection increased significantly to the level of SBP, ABI, TP and TBI.

**Conclusion:**

Compared to resting TcPO2, TcPO2 measured during stimulation improves detection of latent forms of PAD and restenosis/obliterations of previously treated arteries in diabetic foot patients.

**Clinical Trial Registration:**

ClinicalTrials.gov [https://register.clinicaltrials.gov/prs/app/action/SelectProtocol?sid=S0009V7W&selectaction=Edit&uid=U0005381&ts=2&cx=3j24u2], identifier NCT04404699

## Background

In routine foot care, peripheral arterial disease (PAD) is considered an essential diagnostic component in the treatment of diabetic foot (DF). PAD is present in about 50% of patients with DF (1, 2). Macrovascular disease is difficult to detect in many DF patients due to the presence of diabetic sensorimotor neuropathy and medial arterial sclerosis in the lower limbs. Diabetic sensorimotor neuropathy, present in up to 90% of people with diabetes, masks symptoms of PAD due to sensory loss in the lower limbs, while medial arterial sclerosis falsely elevates arterial pressure (3, 4). In addition, autonomic neuropathy, which involves sympathetic denervation affecting the peripheral nerves, leads to the opening of arteriovenous shunts in the microcirculation of the lower limbs as well as alterations in pre-capillary sphincter tone. This group of conditions can result in hypoxia of peripheral tissue even without typical signs of ischaemia, such as cold feet, clinically significant changes in colour and trophic acral lesions (5). Such complications can therefore delay PAD diagnosis, in some cases until the time of DF manifestation (6, 7). Identifying PAD at this late stage doubles the risk of lower limb amputation in patients with DF (8).

The occurrence of PAD not only determines whether a patient is at high risk of developing potential macrovascular comorbidities (9, 10), but also influences DF prognosis and mortality. Therefore, appropriate diagnostic and therapeutic procedures are required if clinicians are to improve early identification of PAD and reduce the risk of amputation and mortality. Both prognosis and risk stratification of DF patients are based on WIfI classifications (11) for wound type and the presence and severity of PAD and infection. The risk of lower limb amputation increases with deeper foot ulcers and, especially, in individuals with advanced ischaemia (ankle-brachial index (ABI) below 0.59 and toe pressure (TP) or transcutaneous oxygen below 39 mm Hg), deep infection or systemic manifestations of infection (10). DF can be stratified based on TcPO2 levels defined in the WIfI classification (11) to assist in wound healing and PAD diagnosis (12).

In this study, we present a novel diagnostic procedure for identifying micro- and macro-circulatory changes in the lower limbs. In diabetic patients, a number of metabolic changes lead to alterations in microcirculation – due to processes such as endothelial dysfunction and circulatory disturbances associated with nutritional supply to the skin – and macrocirculation secondary to accelerated atherosclerosis (9). Diabetes and its late complications such as diabetic neuropathy and mediocalcinosis often limit the accuracy of non-invasive diagnoses of micro- and macro-circulatory changes. Therefore, there is a pressing need to make diagnostic procedures more accurate. The aim of our multicentre study was to refine diagnosis of peripheral arterial disease (PAD) using a new transcutaneous oximetry (TcPO2) stimulation test in patients with DF. Combining TcPO2 measurements with a modification of the Ratschow test, our stimulation test is intended for PAD diagnosis as part of routine clinical practice. We hypothesise that our TcPO2 stimulation test is more accurate at screening PAD compared to resting TcPO2 measurement and reach accuracy of ABI and TBI levels routinely used in clinical practice. Provocative testing can help better differentiate the severity of perfusion abnormalities in patients with TcPO2 levels in the “grey zone”. To our knowledge, this TcPO2 measurement test is the first of its kind to be assessed in patients with PAD and diabetes.

## Methods

### Study Subjects

A total of 79 patients with DF were enrolled as part of the study (mean age – 66.9 ± 10.2 years; diabetes duration – 19.3 ± 12.3 years; HbA1c – 63.9 ± 17.5 mmol/mol; serum creatinine – 105.4 ± 45.6 umol/L; haemoglobin – 133 ± 16 mg/dL; vibration perception threshold – 52 ± 25 V; DF in remission – 19/79 (12.7%); chronic Charcot foot – 9/79 patients (11.4%); DF ulcers according to WIfI classification – W1fI0: 35/79 (44.3%), W1fI1: 5/79 (6.3%), W2fI0: 6/79 (7.6%), W2fI1: 6/79 (7.6), W3fI1: 8/79 (10.1%); all I 1-2). DF was defined as infection, ulceration or destruction of foot tissue in individuals with currently or previously diagnosed diabetes mellitus typically accompanied by neuropathy and/or PAD in the lower extremities (13). All individuals were treated in the outpatient foot clinics of three major Czech diabetes centres (Institute for Clinical and Experimental Medicine, Second Faculty of Medicine, Motol Teaching Hospital, and First Faculty of Medicine, Charles University) from January 2018 to January 2019.

Patients and DF severity were classified based on the WIfI classification. WIfI uses a combination of scores for wound (based on depth of ulcer or extent of gangrene), ischaemia (based on ankle pressure, toe pressure or TcPO2) and foot infection (based on IWGDF/IDSA criteria). These scores provide one-year risk for amputation and one-year benefit for revascularisation, stratified as very low, low, moderate or high. The WIfI classification provides a more holistic wound overview for revascularisation decision-making by extending the associated wound and infection criteria beyond solely perfusion pressure. Whilst WIfI has not been subject to reproducibility assessment in a DFU cohort, it boasts impressive reproducibility in a PAD setting (13). Inclusion criteria were as follows: presence of DF – defined as a foot ulcer associated with neuropathy and peripheral arterial disease in the lower extremities (13) in type 1 or type 2 diabetes mellitus; patients aged 18-70 years classified as WIfI ischaemia 1-2 (based on TcPO2 levels – mean TcPO2 = 40.3 ± 5.8 mm Hg) without previously diagnosed PAD or known PAD based on patient history or previous vascular reconstruction (endovascular or surgical vascular interventions). According to ESC Guidelines, TcPO2 values below 30 mm Hg are demonstrably associated with impaired wound healing, while values ​​above 50 mm Hg are linked to better ulcer healing (12). Therefore, all patients were required to have baseline TcPO2 values ​​of between 30-50 mm Hg at rest.

Exclusion criteria, largely consisting of factors that affect TcPO2 measurements, were as follows: vascular intervention of the evaluated lower limb within 12 months of enrolment; factors possibly influencing oxygen saturation or lower limb movement such as patient immobility or motion impairment of the talocrural joint; vasculitis; heart failure or advanced COPD; severe anaemia (plasma haemoglobin below 8g/dL); hypoperfusion due to shock or cardiac dysfunction; sepsis; massive swelling of the lower limbs of various aetiology (including lymphedoema); active Charcot osteoarthropathy; severe autonomic neuropathy causing orthostatic hypotension, sinus tachycardia or manifesting in other organ systems; critical limb ischaemia of WIfI class ischaemia 3; lower limb claudication below 200m; ulcers of venous insufficiency or combined aetiology; severe diabetic kidney disease (CKD stage 4 or 5) (14).

Patients were treated according to the IWGDF Guidelines on the Prevention and Management of Diabetic Foot Disease, devised to improve diabetes control and detect and treat foot infection and biomechanical changes (15).

Prior to enrolment in the study, each patient signed an informed consent form approved by the local ethics committees of the Institute for Clinical and Experimental Medicine and Thomayer Hospital.

### Assessment of Lower Limb Vascular Status

#### Large Vessels

Assessment of peripheral arterial circulation consisted of foot pulse measurement and ultrasound of the peripheral arteries (16). Systolic blood pressure (SBP) in the peripheral arteries (DPA, Dorsalis pedis artery; PTA, posterior tibial artery) and digital arteries (TP, Toe pressure) was measured using a handheld Doppler ultrasound device with an 8 MHz probe (EDAN SD3 Vascular, DOTmed, NY, USA). The same technique was used to evaluate ankle-brachial indexes (ABI) and toe-brachial indexes (TBI).

Given the frequent inaccuracies associated with ABI, we used colour-coded duplex utrasound (DUS) as the gold standard for PAD detection. DUS was used to assess morphology and flow in the peripheral arteries (4-8 MHz probe, factory default setting, LOGIQ P7, GE HealthCare, USA). Pulse wave correction was set at 70 degrees as standard followed by appropriate adaptation of pulse repetition frequency. Monophasic waves were used to determine the presence of haemodynamically significant stenosis or obliteration with collateralisation. Those arterial lesions modifying pulse waveforms are considered to be clinically hemodynamic significant to reduce dramatically peripheral perfusion.

#### TcPO2

**Figure 1:**
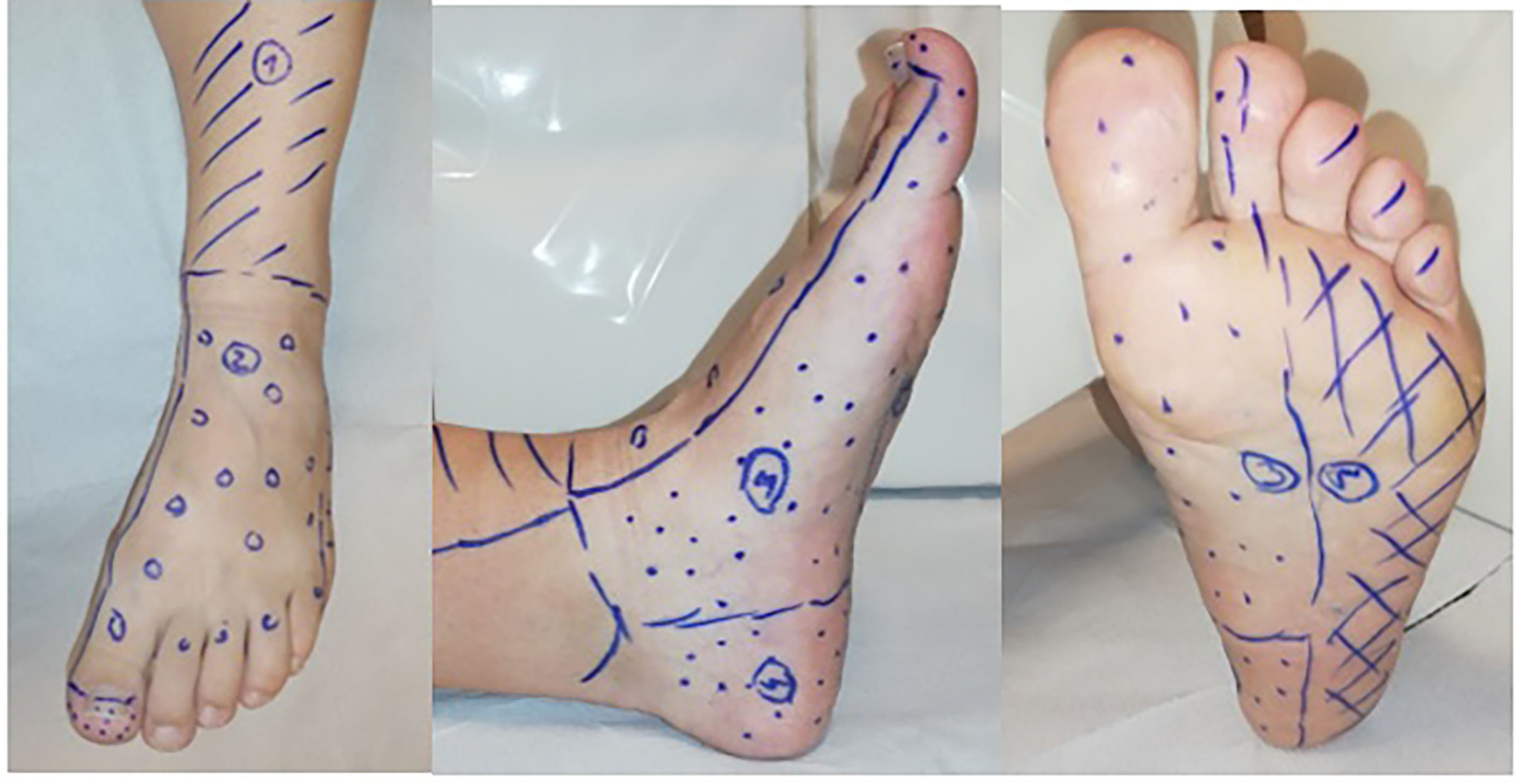
Distribution of angiosomes in the lower limb. 1- Angiosomes supplied by: anterior tibial artery (ATA); 2 - dorsalis pedis artery (DPA); 3 –medial plantar artery; 4 - calcaneal branch of the posterior tibial artery (PTA) (angiosomes 3 and 4 are supplied by the PTA); 5 - lateral plantar artery.

Transcutaneous oxygen pressure (TcPO2) was measured on the Clark electrode, a method used to electrochemically assess partial pressure of oxygen on the surface of the skin (17). A standard probe, consisting of a small chamber containing silver and platinum electrodes with an oxygen-permeable membrane, was heated to 42-45°C for arterialised cutaneous flow, thus increasing oxygen diffusion through the skin *via* local vasodilatation. The results obtained were automatically recalculated to 37°C (17). Prior to measurement, subjects were required to rest in a quiet room maintained at a temperature of 20°C and to abstain from the use of tobacco and alcohol. TcPO_2_ was subsequently measured in respective angiosomes (**Figure 1**) (18, 19).

All centres used the same device for TcPO2 measurement (TINA, Radiometer, Copenhagen). Since photoelectric probes used by other devices result in approximately 15 mm Hg-higher values when measured at rest, electrochemical methods were applied as standard (20).

### TcPO2 Stimulation Test

After TcPO2 measurement of the respective angiosomes (probes placed in angiosomes supplied by DPA and/or PTA) (**Figure 1**) and once a steady resting state was achieved, the modified Ratschow stimulation test was performed (21, 22). The standardised Ratschow test consists of elevating both lower extremities to approximately 50 degrees for 30 seconds. At this stage, a change in skin colour is considered specific to ischaemia. While in the elevated position, the subject continues with dorsal and plantar flexions of the talocrural joints for 90 seconds. Provocation of pain or discoloration at this stage may be a sign of ischaemia. The subject then returns the lower legs to a horizontal position. At this point, physiological changes in skin colour are again monitored. Although once widely used in Western and Central Europe, the technique has now largely become obsolete due to the increased availability of Doppler and duplex ultrasound devices. Active dorsal and plantar flexions of the talocrural joints are mostly constant, having almost no influence on active long-term contractions of the thigh muscles. Moreover, the dependent position the patient returns to remains unchanged.

**Figure 2:**
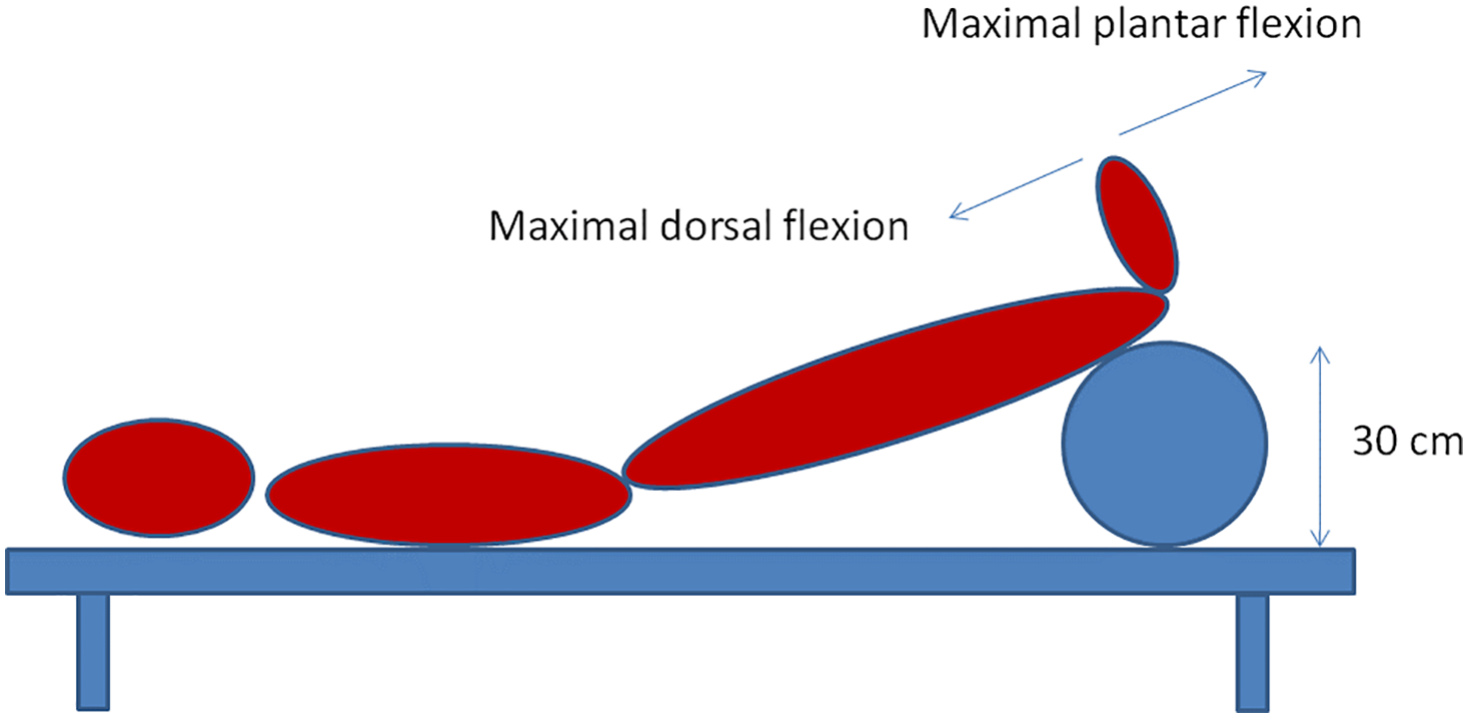
Modified Ratschow test.

Our modification of the Ratschow test involves elevating the lower limbs (30 cm above the bed) followed by rhythmic maximal plantar flexion and extension of the talocrural joints for 2 minutes (**Figure 2**).

Since active elevation of the lower extremities can be problematic in subjects with joint, vertebral column or muscle impairment, we used a cushion with a predefined height for mechanical support (**Figure 2**). TcPO2 was measured continuously throughout. The lower limbs were returned to the horizontal position at the end of the exercise before continuing with TcPO2 measurement. Considering the novel nature of the test and based on our clinical experience, we extended the post-exercise recovery window to at least 8-10 minutes to ensure TcPO2 normalisation. This stimulation test is not time-consuming and lasts approximately 20-25 minutes. It could supplement the standard TcPO2 measurement.

### Evaluation of Large Vessels and TcPO2 Parameters

We assessed 107 angiosomes in 79 patients, dividing them (angiosomes) according to flow type as detected by DUS. In a small minority of patients (8.9% of patients - 7/79), both lower limbs were examined. However, in the majority of patients, only the affected lower limb was examined. We examined 1, maximum 2 angiosomes based on DPA and/or PTA distribution. Angiosomes were divided into two groups: group M (n=60) with verified monophasic flow or obliteration; group T (n=47) with verified triphasic flow. Flow in PTA was detected by the probe located behind the ankle and DPA by probe placed proximally on the instep of the foot. Five angiosomes were excluded from the overall statistical evaluation after DUS detected biphasic arterial flow. All patients with verified biphasic flow were excluded from the overall statistical evaluation.

**Figure 3:**
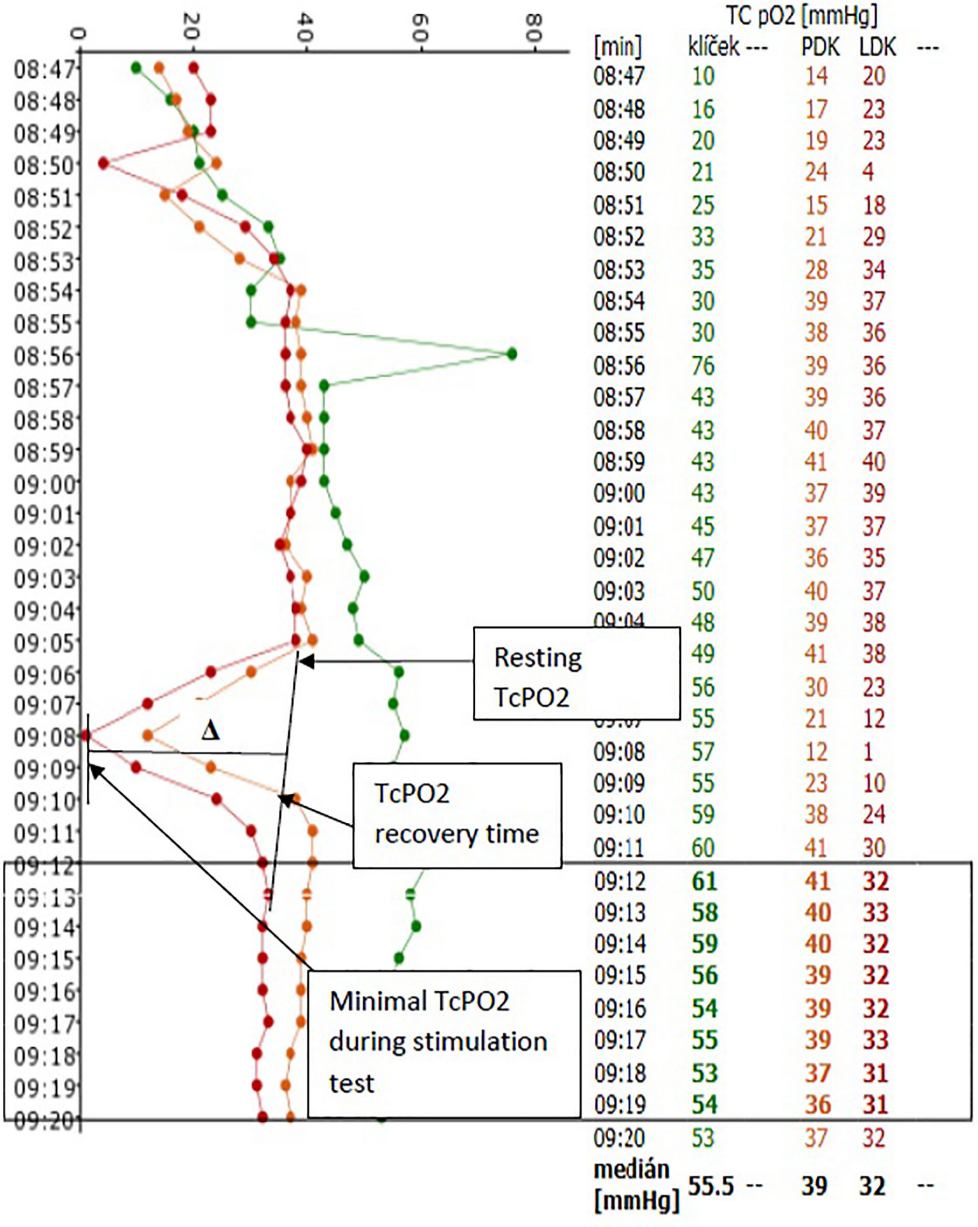
Record of TCPO2 measurement during stimulation test Evaluated parameters: Resting TcPO2; maximal decline of TcPO2 during stimulation test, minimal TcPO2 during stimulation test; TcP02 differences in absolute numbers (△ =resting TcPO2 minus minimal TcPO2 during the stimulation test) and in percentages; TcPO2 recovery time.

We further compared macrocirculation parameters (systolic pressure in DPA and PTA) and Doppler indexes with the ABIs of DPA and PTA, toe pressure (TP) and TBIs between study groups. The following parameters were also compared: resting TcPO2; minimal TcPO2 (23) detected during the stimulation test; delta value (Δ) – defined as resting TcPO2 minus minimal TcPO2 during the stimulation test; percentage decrease in TcPO2 during the stimulation test; TcPO2 recovery time before returning to resting values (**Figure 3**). We also compared correlations of all TcPO2 values to macrocirculation parameters, including sensitivity and specificity, in relation to the presence of PAD based on monophasic arterial flow in the respective artery.

### Statistical Analysis

The Shapiro-Wilk test was used for Gaussian distribution. Gaussian variables were assessed using the t-test. For variables differing from Gaussian distribution, the Mann-Whitney test was applied. For discrete variables, the χ^2^-test of independence with contingency tables were used. To measure relations between variables, the correlation coefficient was used. Sensitivity and specificity were demonstrated using ROC curves. Youden’s J-index was used to determine the optimal cut-point based on ROC analysis. A two-sided p-value of less then 0.05 was considered statistically significant. All calculations were carried out using JMP 11 statistical software (2013) (SAS Institute, Cary, USA).

## Results

Study groups differed significantly with regard to incidence of previously diagnosed PAD (84% of patients in group M vs. 43% of patients in group T; p<0.0001) and the number of patients to have undergone revascularisation procedures (68.4% of patients in group M vs. 26.2% in group T; p<0.0001). The other baseline characteristics were not found to be significantly different (**Table 1**).

**Table 1:** Comparison of basal characteristics between study groups.

Evaluated parameters	Group M (n = 60)	Group T (n = 47)	p-value
Age (years)	68.6 ± 9.9	66.2 ± 9.05	NS
Diabetes duration (years)	19.03 ± 12.6	19.13 ± 11.4	NS
HbA1c (mmol/mol)	61.7 ± 17.2	65.9 ± 14.4	NS
Creatinine serum level (umol/l)	107.8 ± 36	100.2 ± 47.5	NS
Hb (mg/dl)	132.8 ± 17.2	131.6 ± 13.7	NS
Biothesiometer (V)	48 ± 20.4	55.3 ± 27.2	NS

NS, non significant.

During the TcPO2 stimulation test, we observed a significant decrease in TcPO2 in both study groups (from 40.6 to 26.2 mm Hg in group M and from 41.4 to 31.4 mm Hg in group T; both p<0.0001) (**Table 2**). However, the decrease of TcPO2 (Δ) was significantly higher in group M compared to group T (-14.4 ± 9.6 vs. -9.88 ± 8.5 mm Hg; p=0.014). There was also a significant percentage decrease of TcPO2 in group M compared to group T during the stimulation test (-36 ± 25 vs. -24 ± 21%; p=0.007). TcPO2 recovery time was also different, with recovery-to-baseline much higher in group M (446 ± 134 vs. 370 ± 81 seconds; p=0.0005) (**Table 2**).

**Table 2:** Comparison of TcPO2 parameters detected during stimulation test and large vessel parameters between study groups.

Evaluated parameters	Group M (n = 60)	Group T (n = 47)	p-value
Resting TcPO2 (mm Hg)	40.6 ± 5.9	41.4 ± 5.5	NS
Minimal TcPO2 detected during stimulation test (mm Hg)	26.2 ± 11.1	31.4 ± 9.4	p=0.01
TcPO2 after stimulation test	40.3 ± 8.4	41.8 ± 6.7	NS
Δ of TcPO2 (= resting minus minimal TcPO2)	14.4 ± 9.6	9.98 ± 8.5	p=0.014
Percentage of TcPO2 decrease (%)	36 ± 25	24 ± 21	p=0.007
Duration of TcPO2 recovery (s)	446 ± 134	370 ± 81	p=0.0005
Systolic blood pressure in DPA (mm Hg)	149 ± 62	183 ± 44	p=0.007
Systolic blood pressure in PTA (mm Hg)	138 ± 68	181 ± 50	p=0.001
ABI-DPA	0.93 ± 0.4	1.2 ± 0.3	p<0.0001
ABI-PTA	0.9 ± 0.4	1.2 ± 0.3	p=0.0004
TP (mm Hg)	59.9 ± 19.9	89.5 ± 30.8	p<0.0001
TBI	0.39 ± 0.11	0.62 ± 0.21	p<0.0001

n, number; TcPO2, transcutaneous oxygen measurement; mm Hg, millimetres of mercury; Δ, delta difference; %, percentage; NS, non-significant; s, seconds; DPA, dorsalis pedis artery; PTA, posterior tibial artery; ABI, ankle-brachial index; TP, toe pressure; TBI, toe-brachial index.

Both systolic ankle pressure, TP and their respective indexes (ABI-DPA, ABI-PTA and TBI) were significantly lower in group M compared to group T (**Table 2**). We found positive correlations of ABI-DPA, ABI-PTA, TP and TBI. While we observed minimal TcPO2 values achieved during exercise (**Table 2**), we found negative correlations of TP and TBI to Δ TcPO2, the percentage decrease of TcPO2, and TcPO2 recovery time (**Table 3**).

**Table 3:** Correlation between large-vessel and TcPO2 stimulation parameters.

Large-vessel parameters	TcPO2 parameters	r	p-value
Systolic blood pressure in DPA (mm Hg)	Minimal TcPO2	0.0746	NS
	Δ of TcPO2	-0.05360	NS
	% decrease of TcPO2	-0.0678	NS
	Duration of TcPO2 recovery	-0.2045	0.0784
Systolic blood pressure in PTA (mm Hg)	Minimal TcPO2	0.0916	NS
	Δ of TcPO2	-0.0446	NS
	% decrease of TcPO2	-0.0703	NS
	Duration of TcPO2 recovery	-0.1602	NS
ABI-DPA	Minimal TcPO2	0.2634	0.0175
	Δ of TcPO2	-0.1758	NS
	% decrease of TcPO2	-0.2148	0.0542
	Duration of TcPO2 recovery	-0.2096	0.0604
ABI-PTA	Minimal TcPO2 values	0.2208	0.0297
	Δ of TcPO2	-0.1345	NS
	% decrease of TcPO2	-0.1758	0.0850
	Duration of TcPO2 recovery	-0.2303	0.0233
TP (mm Hg)	Minimal TcPO2	0.2864	0.0127
	Δ of TcPO2	-0.3655	0.0013
	% decrease of TcPO2	-0.3619	0.0014
	Duration of TcPO2 recovery	-0.4113	0.0002
TBI	Minimal TcPO2	0.3533	0.0017
	Δ of TcPO2	-0.4051	0.0003
	% decrease of TcPO2	-0.4141	0.0002
	Duration of TcPO2 recovery	-0.4747	<0.0001

n, number; r, correlation coefficient; DPA, dorsalis pedis artery; PTA, posterior tibial artery; ABI, ankle-brachial index; mm Hg, millimetres of mercury; TP, toe pressure; TBI, toe-brachial index; TcPO2, transcutaneous oxygen measurement; mm Hg, millimetres of mercury; Δ, delta difference; %, percentage; NS, non-significant.

**Figure 4:**
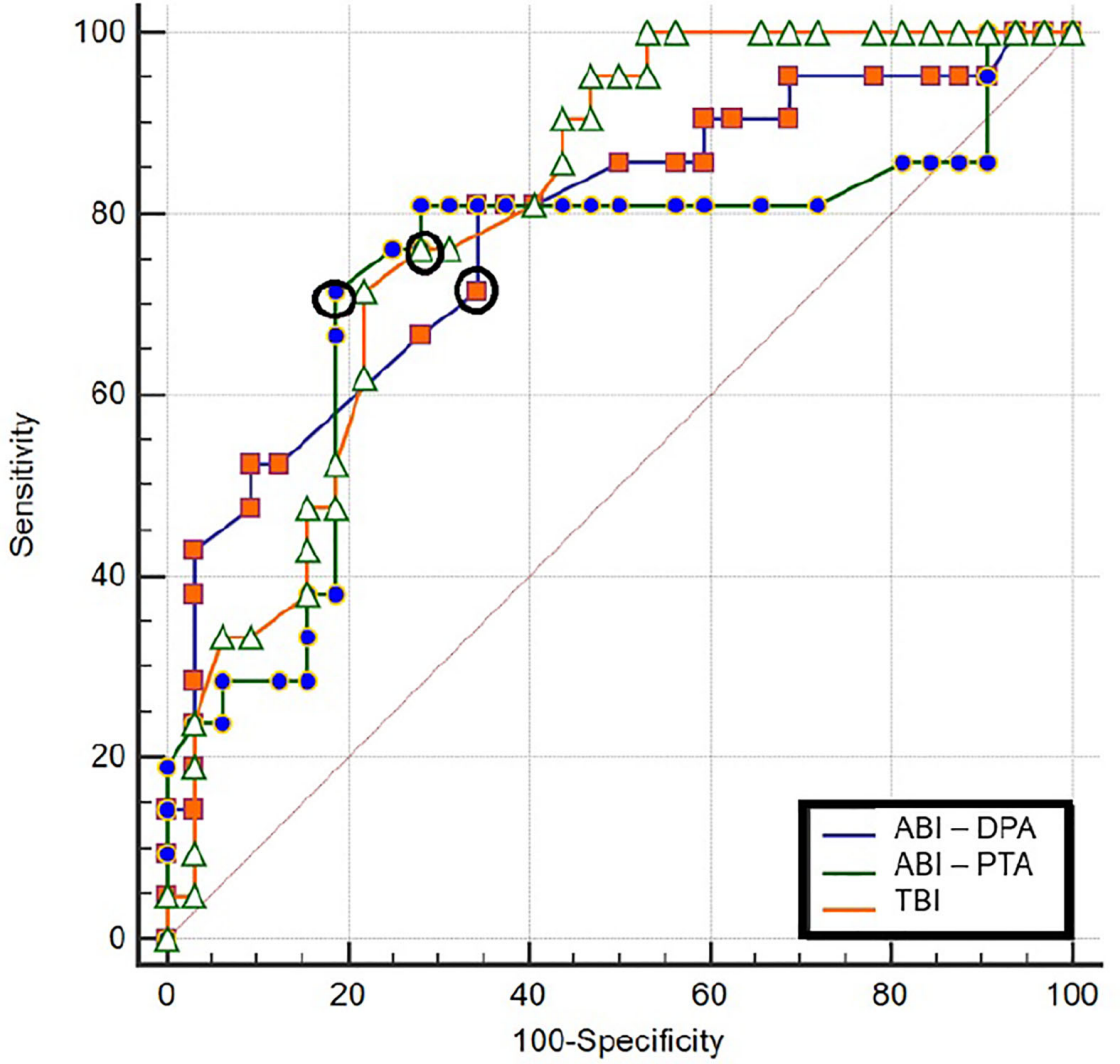
Sensitivity and specificity of individual macrovascular assessment in relation to monophasic arterial flow supplying the relevant angiosome. ABI, ankle-brachial index; DPA, dorsalis pedis artery; PTA, posterior tibial artery; TBI, toe-brachial index; p, significance; CI, confidence interval; mm, Hg-millimetres of mercury; the highest sensitivity and specificity for individual measurements are highlighted in bold circles.

**Figure 5:**
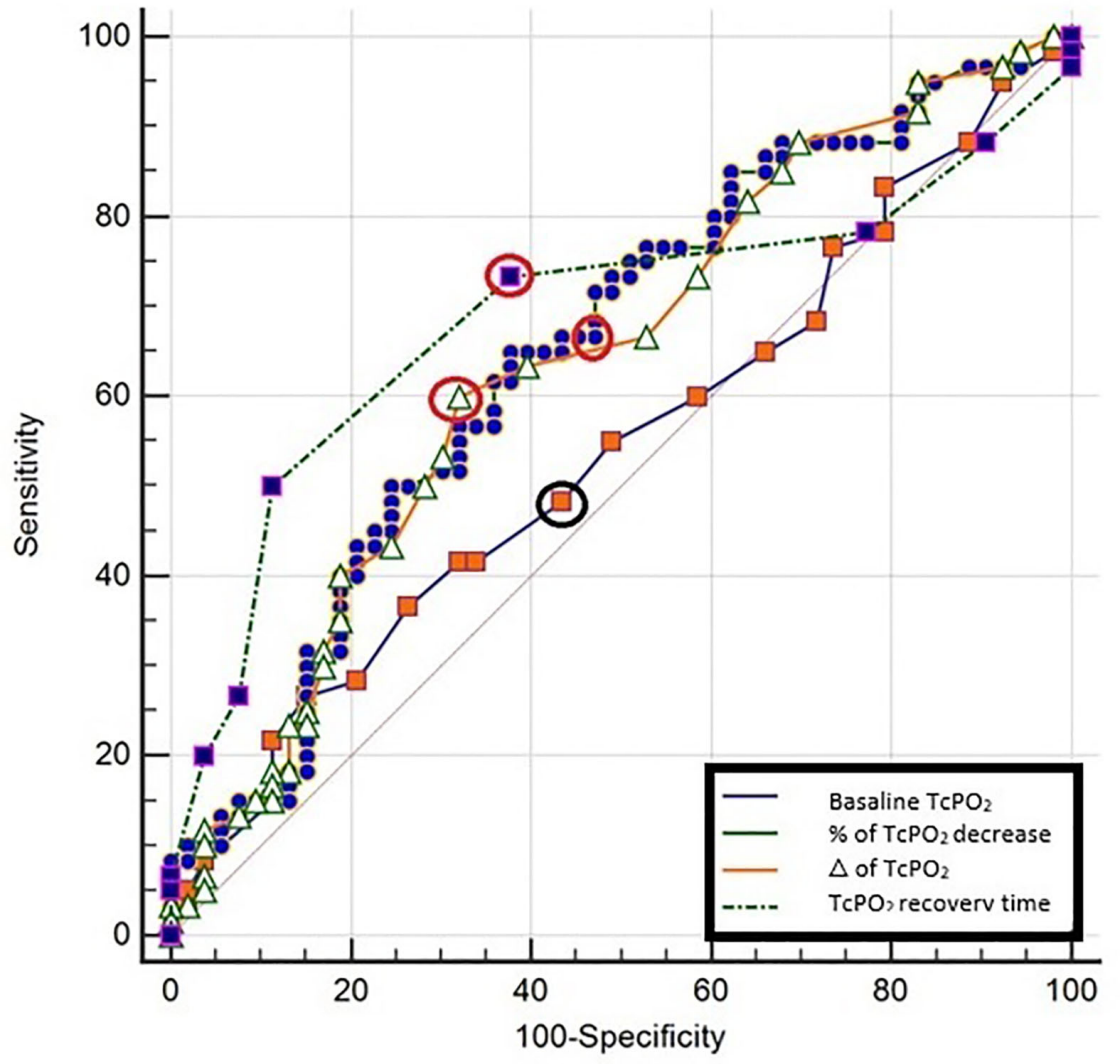
Sensitivity and specificity of microvascular assessment and TcPO2 stimulation parameters in relation to monophasic arterial flow supplying the relevant angiosome. TcPO2, transcutaneous oxygen measurement;△ -difference of TcPO2 counted as resting TcPO2 minus minimal TcPO2 detected during the stimulation test; %-percentage; p-significance; CI, confidence interval; mmHg, millimetres of mercury; the highest sensitivity and specificity for individual measurements are highlighted in bold circles.

Sensitivity and specificity of macrocirculation parameters (systolic ankle pressure, TP and their indexes ABI-DPA, ABI-PTA and TBI) in relation to monophasic flow ranged between 69-78% and 64-78%, respectively (**Table 4** and **Figure 4**). For individual macrocirculation parameters considered decisive according to WIfI classification such as an ABI ≤ 0.4, sensitivity was below 10% and 8%, with specificity at 100% and 100%, respectively; for an ABI ≤ 0.8, sensitivity was 35.1% and 42.9%, with specificity at 97.73% and 77.1%, respectively. Similarly, TP ≤ 30 mm Hg sensitivity was 10.3% and specificity 97.2%, while TP ≤ 60 mm Hg sensitivity was 69.2% and specificity 77.8% (**Table 4** and **Figure 4**). The sensitivity and specificity of resting TcPO2 ​(<39 mm Hg) in relation to monophasic flow was 48% and 57%, respectively (**Table 5**). After stimulation, however, the informative value of the TcPO2 parameters measured (Δ TcPO2 and the percentage decrease of TcPO2) increased dramatically, with sensitivity reaching 60-65% and specificity 62-68% (**Table 5** and **Figure 5**). During the stimulation test, the highest sensitivity and specificity achieved was for TcPO2 recovery time (above 360 ​s; sensitivity at 73.3% and specificity at 68.1%) (**Figure 5**).

**Table 4:** Sensitivity and specificity of individual macrovascular assessment in relation to monophasic arterial flow supplying the relevant angiosome.

Evaluated parametersDetermined values	SBP DPA – sensitivity (95% CI)		SBP DPA – specificity (95% CI)	Evaluated parameter	SBP PTA – sensitivity (95% CI)	SBP PTA – specificity (95% CI)	Evaluated parameter	SBP TP – sensitivity (95% CI)	SBP TP – specificity (95% CI)
**<20 mm Hg**	0.0% (0.0-8.8)		100% (90.0-100)	**<0 mm Hg**	0% (0-6.7)	100% (91.0-100)	**<15 mm Hg**	0% (0-9.0)	100% (90.3-100)
**≤75**	7.5% (1.6-20.4)		100% (90.0-100)	**≤20**	9.43% (3.1-20.7)	100% (91.0-100)	**≤20**	2.56% (0.06-13.5)	97.22% (85.5-99.9)
**≤80**	10% (2.8-23.7)		97.14% (85.1-99.9)	**≤70**	11.32% (4.3-23.0)	97.44% (86.5-99.9)	**≤30**	10.26% (2.9-24.2)	97.22% (85.5-99.9)
**≤100**	27.5% (14.6-43.9)		97.14% (85.1-99.9)	**≤90**	20.75% (10.8-34.1)	97.44% (86.5-99.9)	**≤40**	12.82% (4.3-27.4)	88.89% (73.9-96.9)
**≤110**	27.5% (14.6-43.9)		94.29% (80.8-99.3)	**≤100**	24.53% (13.8-38.3)	89.74% (75.8-97.1)	**≤47**	25.64% (13-42.1)	88.89% (73.9-96.9)
**≤116**	32.5% (18.6-49.1)		94.29% (80.8-99.3)	**≤104**	28.3% (16.8-42.3)	89.74% (75.8-97.1)	**≤50**	33.33% (19.1-50.2)	83.33% (67.2-93.6)
**≤120**	35% (20.6-51.7)		88.57% (73.3-96.8)	**≤116**	41.51% (28.1-55.9)	82.05% (66.5-92.5)	**≤55**	46.15% (30.1-62.8)	83.33% (67.2-93.6)
**≤130**	47.5% (31.5-63.9)		88.57% (73.3-96.8)	**≤130**	56.6% (42.3-70.2)	74.36% (57.9-87.0)	**≤60**	**69.23% (52.4-83)**	**77.78% (60.8-89.9)**
**≤140**	55% (38.5-70.7)		80% (63.1-91.6)	**≤140**	56.6% (42.3-70.2)	71.79% (55.1-85.0)	**≤64**	69.23% (52.4-83)	75% (57.8-87.9)
**≤142**	57.5% (40.9-73.0)		80% (63.1-91.6)	**≤142**	58.49% (44.1-71.9)	71.79% (55.1-85.0)	**≤70**	74.36% (57.9-87.0)	63.89% (46.2-79.2)
**≤150**	62.5% (45.8-77.3)		74.29% (56.7-87.5)	**≤150**	62.26% (47.9-75.2)	69.23% (52.4-83.0)	**≤75**	76.92% (60.7-88.9)	63.89% (46.2-79.2)
**≤156**	65% (48.3-79.4)		74.29% (56.7-87.5)	**≤160**	64.15% (49.8-76.9)	69.23% (52.4-83.0)	**≤80**	87.18% (72.6-95.7)	55.56% (38.1-72.1)
**≤160**	**70% (53.5-83.4)**		**71.43% (53.7-85.4)**	**≤170**	**69.81% (55.7-81.7)**	**64.1% (47.2-78.8)**	**≤85**	92.31% (79.1-98.4)	52.78% (35.5-69.6)
**≤180**	70% (53.5-83.4)		54.29% (36.6-71.2)	**≤180**	73.58% (59.7-84.7)	43.59% (27.8-60.4)	**≤90**	94.87% (82.7-99.4)	33.33% (18.6-51)
**≤190**	75% (58.8-87.3)		48.57% (31.4-66)	**≤190**	75.47% (61.7-86.2)	38.46% (23.4-55.4)	**≤100**	97.44% (86.5-99.9)	22.22% (10.1-39.2)
**≤200**	75% (58.8-87.3)		37.14% (21.5-55.1)	**≤200**	77.36% (63.8-87.7)	35.9% (21.2-52.8)	**≤110**	100% (91-100)	19.44% (8.2-36)
**≤220**	85% (70.2-94.3)		11.43% (3.2-26.7)	**≤220**	92.45% (81.8-97.9)	15.38% (5.9-30.5)	**≤170**	100% (91-100)	0% (0-9.7)
	**Significance p=0.0032**		**Youden’s J index 0.414**		**Significance p=0.0032**	**Youden’s J index 0.3391**		**Significance p<0.0001**	**Youden’s J index 0.4701**
	**AUC 0.687**		**95% confidence interval 0.569 to 0.789**		**AUC 0.667**	**95% confidence interval 0.561 to 0.762**		**AUC 0.762**	**95% confidence Interval 0.649 to 0.853**
**Evaluated parameters** **Determined** **values**		**ABI DPA – sensitivity (95% CI)**	**ABI DPA – specificity (95% CI)**	**Evaluated parameter**	**ABI PTA– sensitivity (95% CI)**	**ABI PTA– specificity (95% CI)**	**Evaluated parameter**	**TBI – sensitivity (95% CI)**	**TBI – Specificity (95% CI)**
**<0.18**		0.0% (0.0-9.5)	100% (92-100)	**<0**	0.0% (0.0-7.3)	100% (92.6-100)	**<0.13**	0.0% (0.0-8.8)	100% (90.3-100)
**≤0.58**		8.11% (1.7-21.9)	100% (92-100)	**≤0.47**	10.2% (3.4-22.2)	100% (92.6-100)	**≤0.23**	10% (2.8-23.7)	97.2% (85.5-99.9)
**≤0.62**		8.11% (1.7-21.9)	97.73% (88-99.9)	**≤0.54**	12.24% (4.6-24.8)	97.92% (88.9-99.9)	**≤0.31**	30% (16.6-46.5)	91.67% (77.5-98.2)
**≤0.82**		35.14% (20.2-52.5)	97.73% (88-99.9)	**≤0.62**	16.33% (7.3-29.7)	95.83% (85.7-99.5)	**≤0.34**	40% (24.9-56.7)	86.11% (70.5-95.3)
**≤0.83**		37.84% (22.5-55.2)	93.18% (81.3-98.6)	**≤0.7**	28.57% (16.6-43.3)	89.58% (77.3-96.5)	**≤0.4**	52.5% (36.1-68.5)	77.78% (60.8-89.9)
**≤0.9**		40.54% (24.8-57.9)	90.91% (78.3-97.5)	**≤0.8**	42.86% (28.8-57.8)	77.08% (62.7-88)	**≤0.43**	72.5% (56.1-85.4)	75% (57.8-87.9)
**≤0.933**		48.65% (31.9-65.6)	90.91% (78.3-97.5)	**≤0.9**	55.1% (40.2-69.3)	70.83% (55.9-83)	**≤0.47**	**77.5% (61.5-89.2)**	**75% (57.8-87.9)**
**≤0.96**		48.65% (31.9-65.6)	86.36% (72.6-94.8)	**≤1.01**	**69.39% (54.6-81.7)**	**68.75% (53.7-81.3)**	**≤0.5**	85% (70.2-94.3)	58.33% (40.8-74.5)
**≤1**		**75.68% (58.8-88.2)**	**68.18% (52.4-81.4)**	**≤1.06**	73.47% (58.9-85.1)	62.5% (47.4-76)	**≤0.53**	92.5% (79.6-98.4)	55.56% (38.1-72.1)
**≤1.05**		78.38% (61.8-90.2)	63.64% (47.8-77.6)	**≤1.17**	75.51% (61.1-86.7)	47.92% (33.3-62.8)	**≤0.57**	95% (83.1-99.4)	52.78% (35.5-69.6)
**≤1.1**		78.38% (61.8-90.2)	56.82% (41-71.7)	**≤1.22**	81.63% (68-91.2)	45.83% (31.4-60.8)	**≤0.6**	97.5% (86.8-99.9)	47.22% (30.4-64.5)
**≤1.16**		83.78% (68-93.8)	52.27% (36.7-67.5)	**≤1.26**	85.71% (72.8-94.1)	41.67% (27.6-56.8)	**≤1.13**	100% (91.2-100)	0% (0-9.7)
**≤1.3**		86.49% (71.2-95.5)	31.82% (18.6-47.6)	**≤1.4**	85.71% (72.8-94.1)	20.83% (10.5-35)			
**≤1.38**		89.19% (74.6-97)	22.73% (11.5-37.8)	**≤1.52**	93.88% (83.1-98.7)	8.33% (2.3-20)			
**≤1.52**		97.3% (85.8-99.9)	6.82% (1.4-18.7)						
		**Significance p<0.0001**	**Youden’s J index 0.4386**		**Significance p=0.0009**	**Youden’s J index 0.3814**		**Significance p<0.0001**	**Youden’s J index 0.5250**
		**AUC 0.768**	**95% confidence interval 0.660 to 0.854**		**AUC 0.683**	**95% confidence interval 0.581 to 0.774**		**AUC 0.798**	**95% confidence interval 0.690 to 0.881**

SBP, systolic blood pressure; DPA, dorsalis pedis artery; PTA, posterior tibial artery; TP, toe pressure; p, significance; CI, confidence interval, mm Hg, millimetres of mercury; AUC, area under the ROC curve; ABI, ankle-brachial index; TBI, toe-brachial index; p, significance; the highest sensitivity and specificity for individual measurements are highlighted in bold.

**Table 5:** Sensitivity and specificity of microvascular assessment and TcPO2 parameters in relation to monophasic arterial flow supplying the relevant angiosome.

Evaluated parameters Determined values	Resting TcPO2 sensitivity (95% CI)	Resting TcPO2 specificity (95% CI)	Evaluated parameter	Δ of TcPO2 sensitivity (95% CI)	Δ of TcPO2 specificity (95% CI)	Evaluated parameter	% decrease of TcPO2 sensitivity (95% CI)	% decrease of TcPO2 specificity (95% CI)
**<30 mm Hg**	0.0% (0.0-6.0)	100% (93.3-100)	**≤0 mm Hg**	100.0% (94-100)	1.89% (0.05-10.1)	**≤0**	100.0% (94.0-100)	1.89% (0.05-10.1)
**≤33**	15% (7.1-26.6)	88.7% (77-95.7)	**>0**	98.33% (91.1-100)	5.66% (1.2-15.7)	**>0**	98.33% (91.1-100)	5.6% (1.2-15.7)
**≤35**	**26.67% (16.1-39.7)**	**84.91% (72.4-93.3)**	**>3**	91.67% (81.6-97.2)	16.98% (8.1-29.8)	**>5**	96.67% (88.5-99.6)	11.32% (4.3-23)
**≤38**	41.7% (29.1-55.1)	67.92% (53.7-80.1)	**>5**	85% (73.4-92.9)	32.08% (19.9-46.3)	**>8**	91.67% (81.6-97.2)	18.87% (9.4-32)
**≤40**	48.33% (35.2-61.6)	56.6% (42.3-70.2)	**>8**	66.67% (53.3-78.3)	47.17% (33.3-61.4)	**>12**	88.33% (77.4-95.2)	32.08% (19.9-46.3)
**≤43**	65% (51.6-76.9)	33.96% (21.5-48.3)	**>10**	**60% (46.5-72.4)**	**67.92% (53.7-80.1)**	**>17.8**	76.67% (64-86.6)	39.62% (26.5-54)
**≤45**	76.67% (64-86.6)	26.42% (15.3-40.3)	**>13**	43.33% (30.6-56.8)	75.47% (61.7-86.2)	**>20**	66.67% (53.3-78.3)	52.83% (38.6-66.7)
**≤48**	88.33% (77.4-95.2)	11.32% (4.3-23)	**>15**	35% (23.1-48.4)	81.13% (68-90.6)	**>23**	**65% (51.6-76.9)**	**62.26% (47.9-75.2)**
**≤50**	98.33% (91.1-100)	1.89% (0.05-10.1)	**>18**	25% (14.7-37.9)	84.91% (72.4-93.3)	**>25**	61.67% (48.2-73.9)	62.26% (47.9-75.2)
**≤51**	100% (94-100)	0% (0-6.7)	**>20**	23.33% (13.4-36)	86.79% (74.7-94.5)	**>30**	50% (36.8-63.2)	73.58% (59.7-84.7)
			**>24**	15% (7.1-26.6)	88.68% (77.0-95.7)	**>35**	43.33% (30.6-56.8)	79.25% (65.9-89.2)
			**>29**	11.67% (4.8-22.6)	96.23% (87-99.5)	**>40**	31.67% (20.3-45)	84.91% (72.4-93.3)
			**>35**	3.33% (0.4-11.5)	98.11% (89.9-100)	**>60**	15% (7.1-26.6)	86.79% (74.7-94.5)
			**>39**	0% (0-6)	100% (93.3-100)	**>100**	0% (0-6.0)	100% (93.3-100)
	**Significance NS**	**Youden’s J index 0.1157**		**Significance p=0.0075**	**Youden’s J index 0.2792**		**Significance p=0.0037**	**Youden’s J index 0.2726**
	**AUC 0.541**	**95% confidence interval 0.445 to 0.635**		**AUC 0.640**	**95% confidence interval 0.545 to 0.728**		**AUC 0.651**	**95% confidence interval 0.556 to 0.738**

TcPO2, transcutaneous oxygen measurement; Δ, difference of TcPO2 counted as resting TcPO2 minus minimal TcPO2 detected during stimulation test; %, percentage; p, significance; CI, confidence interval, mm Hg, millimetres of mercury; AUC, area under the ROC curve; the highest sensitivity and specificity for individual measurements are highlighted in bold.

## Discussion

In routine podiatry practice, TcPO2 is used to determine the state of microcirculation and estimate the probability of wound healing (24). The literature suggests that TcPO2 has a reliable predictive value in the prognosis of diabetic foot ulcer (DFU) healing (with sensitivity at 72% and specificity at 86%) (25). There is a higher probability of wound healing in patients with TcPO​​2 values above 40 mm Hg (26). However, in many cases, TcPO2 does not reveal the true state of macrocirculation. Recently, TcPO2 was added to the WIfI classification system. Non-ischaemia is defined as a TcPO2 value above 60 mm Hg, with mild ischaemia between 40 and 59 mm Hg. Patients with TcPO2 values between 30 and 39 mm Hg typically suffer from moderate PAD and those with TcPO2 <30 mm Hg from critical limb-threatening ischaemia. The European Society for Vascular Surgery (ESVS) Guidelines define the impairment of circulation status as a TcPO2 ​​value below 40 mm Hg and state that alteration of large vessels is frequently detected in patients with TcPO2 ​​values below 40 mm Hg (10). Clinically, however, healing processes can alter in patients with TcPO2 ​​values above 30 or 40 mm Hg. Therefore, to establish the presence of PAD, such patients should either undergo non-invasive examination or treatment involving the modification of existing non-invasive methods.

There are notable limitations to the use of standard non-invasive PAD diagnostics in routine clinical practice. Many patients with DF do not display classic PAD symptoms (coldness/acral lividosis, colour changes, claudication, resting ischaemic pain or weak/absent peripheral pulsation), most often due to distal sensorimotor neuropathy (2, 27). Doppler examination of ABI and TP is often inapplicable in cases of medial sclerosis (10), skin lesions that contraindicate the use of instruments, and the absence of toes. Also, ultrasound examination of lower limb arteries can be complicated by the presence of arterial calcifications, especially in distal vessels (28).

TcPO2 is a well-established method for assessing tissue perfusion/oxygenation. The use of other methods such as laser Doppler and laser speckle imaging are less common. TcPO2 devices are relatively inexpensive and widely used in diabetic, vascular and surgery clinics throughout the Czech Republic. Therefore, any potential improvements in examination protocols are likely to be easily implemented. According to recent guidelines, arterial flow should be examined, especially in patients with diabetes, using several non-invasive methods or by modifying existing procedures to increase the predictive value of PAD (29). However, there are occasional discrepancies between individual diagnostic findings. For instance, although high TcPO2 levels typically indicate a satisfactory state of microcirculation, some DUS results clearly prove severe atherosclerotic involvement in macrocirculation. In this study, we used a modified TcPO2 procedure commonly used in podiatry practice to refine PAD diagnosis (including latent PAD and restenosis/obliteration following revascularisation) in patients with DF. In addition to standard TcPO2 examination (30), we used a modified Ratschow stimulation test (31), which augments TcPO2 measurements through exercise, similar to other methods described by Audonnet (32), Abraham (33) and Kovacseva (34). Previous studies have shown that exercise TcPO2 stimulation tests may be better at detecting proximal ischaemia (32) and peripheral arterial disease (34). For the stimulation tests performed in the above studies, the treadmill was used as the standard exercise equipment. However, this mode of exercise is not recommended in DF patients who (i) have claudication associated with peripheral sensorimotor neuropathy, (ii) are required to off-load affected limbs, (iii) have been previously amputated, or (iv) are prone to physical instability. To provide a stimulus that would not load the lower extremities, we performed a modified Ratschow test. To date, this type of TcPO2 stimulation test has yet to be performed in patients with suspected PAD, diabetes or DF where the localisation of lesions is likely to be peripheral.

During the TcPO2 stimulation test, once patients attained a steady state verified by resting TcPO2 measurements, the lower limbs were elevated followed by maximal plantar and dorsal flexions. TcPO2 levels decreased in nearly all of the patients evaluated. However, in the group exhibiting monophasic flow or proven arterial obliteration in angiosomes, the decrease in TcPO2 was more noticeable (>14 mm Hg) than in patients with triphasic flow. These findings are consistent with the results of the Audonnet study, which confirmed proximal arterial stenosis and a reduction in TcPO2 of 15 mm Hg during a stimulation walking test (32). In our study, we found greater differences between groups in relation to the percentage of TcPO2 reduction and TcPO2 recovery time. In cases where arteries were obliterated or significantly stenotic, TcPO2 recovery lasted at least 7 minutes. However, TcPO2 recovery time during the TcPO2 stimulation test was significantly shorter (at around 6 minutes) in patients with physiological triphasic flow.

In contrast, to prove correlations with ABI and TBI, resting TcPO2 did not correlate with impairment of large vessels, as confirmed by ultrasound findings. Indeed, the sensitivity of resting TcPO2 ​​(approx. 48%) was very low for PAD diagnosis. However, when TcPO2 measurements were augmented by the modified Ratchow test, the sensitivity and specificity of all TcPO2 stimulation parameters used to detect ischaemic lesions increased significantly by up to 25% and 11%, respectively. Thus, the parameters for the TcPO2 stimulation test were comparable to the sensitivity and specificity of systolic pressure values for DPA, PTA (70% and 64-71%, respectively), ABIs (76% and 69%, respectively), TPs and TBIs (70-78% and 75-78%, respectively). According to our data analysis, PAD caused by arterial stenosis/obliteration occurred most frequently in patients with ABI ≤1.0, TP <60 mm Hg and TBI <0.47. These findings correspond mostly with the WIfI classification of DF patients. In patients with mild PAD of the lower limbs and with ABI <0.8, sensitivity and specificity was 35-43% and 77-97%, respectively; in patients with severe PAD and ABI <0.6, sensitivity and specificity was 8-16% and 96-97%, respectively. Our findings for TP are also in line with recommendations (10).

The study has some limitations. There is a possibility that the patients we examined displayed either exhibited limited joint mobility or were not fully compliant with the foot exercises prescribed, two valid criteria for exclusion. However, these minor changes are not likely to have considerably interfered with results, since joint movement in different ranges does not affect the maximal effort exerted by the lower limb muscles when tensing and contracting (35). Indeed, maximal muscle activity in conjunction with leg elevation can lead to lower perfusion of acral and muscular tissues due to gravity. Further, we eliminated other factors that may have affected our final TcPO2 results by performing the test under tightly defined conditions, e.g. providing a calm environment and a sufficiently lengthy examination time, setting clearly defined leg elevations, maintaining an unvarying temperature in the room, and ensuring accurate probe placement (18, 36). Another limitation could be a relatively small number of enrolled patients or evaluated angiosomes but based on study power analysis all assessed parameters had in our study sufficient power – f.e. at least for Δ of TcPO2 was power of the study for p=0.05 -85.6% and for % decrease of TcPO2 85.6%; respectively.

## Conclusion

In conclusion, we found no association between resting TcPO2 and significant stenosis or obliteration of arteries supplying angiosomes, as verified by DUS wave analysis. The highest sensitivity and specificity for detecting stenotic/obliterated arteries were observed in cases where TcPO2 decreased (Δ) by more than 10 mm Hg or by 23% in comparison to resting values. However, in terms of routine podiatry care, we assume the most important parameter to be TcPO2 recovery time. Recovery lasting more than 6 minutes correlated with a high probability of haemodynamically significant stenosis or obliteration in the arteries supplying the respective angiosomes. TcPO2 stimulation parameters not only correlated with indicators used for detecting alterations in large vessels, but also dramatically increased the sensitivity and specificity of TcPO2 measurement in relation to PAD detection. Therefore, this type of TcPO2 stimulation test is likely to be more accurate than the more common resting TcPO2 measurement at detecting latent forms of PAD or restenosis/obliteration in DF patients with previous arterial intervention where macrocirculation parameters cannot always be properly assessed. As such, our novel test represents an easier and quicker non-invasive method of PAD assessment, helping to delay progression of local findings due to asymptomatic PAD in patients with DF.

## Data Availability Statement

The raw data supporting the conclusions of this article will be made available by the authors, without undue reservation.

## Ethics Statement

The studies involving human participants were reviewed and approved by Ethics committees of the Institute for Clinical and Experimental Medicine and Thomayer Hospital. The patients/participants provided their written informed consent to participate in this study.

## Author Contributions

JM and KR contributed to the study protocol and designed and reviewed the manuscript. PP, MF, VW, MD, and JH researched the data and reviewed the manuscript. EJ and AJ reviewed/edited the manuscript. RB reviewed the manuscript and VL performed the statistical analysis. All authors were equally entitled to query any aspect of the data, either directly or through independent analysis. AJ is the guarantor of the trial, retains full access to all data and assumes responsibility for the integrity and accuracy of the data analysed. All authors contributed to the article and approved the submitted version.

## Funding

This study was supported by the Ministry of Health of the Czech Republic through grant NU20-01-00078 and its conceptual development of research organizations programme (Institute for Clinical and Experimental Medicine – IKEM, IN 00023001).

## Conflict of Interest

The authors declare that the research was conducted in the absence of any commercial or financial relationships that could be construed as a potential conflict of interest.

## Publisher’s Note

All claims expressed in this article are solely those of the authors and do not necessarily represent those of their affiliated organizations, or those of the publisher, the editors and the reviewers. Any product that may be evaluated in this article, or claim that may be made by its manufacturer, is not guaranteed or endorsed by the publisher.
